# miR-21 increases c-kit^+^ cardiac stem cell proliferation *in vitro* through PTEN/PI3K/Akt signaling

**DOI:** 10.7717/peerj.2859

**Published:** 2017-01-05

**Authors:** Bei Shi, Wenwen Deng, Xianping Long, Ranzun Zhao, Yan Wang, Wenming Chen, Guanxue Xu, Jin Sheng, Dongmei Wang, Song Cao

**Affiliations:** 1 Department of Cardiology, Affiliated Hospital of Zunyi Medical College, Zunyi, Guizhou, China; 2 Department of Anesthesiology, Zunyi Medical College, Zunyi, Guizhou, China; 3 Guizhou Key Laboratory of Anesthesia and Organ Protection, Zunyi Medical College, Zunyi, Guizhou, China

**Keywords:** c-kit^+^ cardiac stem cell, microRNA-21, Proliferation, PTEN, PI3K-Akt pathway, Ischemic cardiomyopathy

## Abstract

The low survival rate of cardiac stem cells (CSCs) in the ischemic myocardium is one of the obstacles in ischemic cardiomyopathy cell therapy. The MicroRNA (miR)-21 and one of its target protein, the tensin homolog deleted on chromosome ten (PTEN), contributes to the proliferation of many kinds of tissues and cell types. It is reported that miR-21 promotes proliferation through PTEN/PI3K/Akt pathway, but its effects on c-kit^+^ CSC remain unclear. The authors hypothesized that miR-21 promotes the proliferation in c-kit**^+^** CSC, and evaluated the involvement of PTEN/PI3K/Akt pathway *in vitro*. miR-21 up-regulation with miR-21 efficiently mimics accelerated cell viability and proliferation in c-kit**^+^** CSC, which was evidenced by the CCK-8, EdU and cell cycle analyses. In addition, the over-expression of miR-21 in c-kit**^+^** CSCs notably down-regulated the protein expression of PTEN although the mRNA level of PTEN showed little change. Gain-of-function of miR-21 also increased the phosphor-Akt (p-Akt) level. Phen, the selective inhibitor of PTEN, reproduced the pro-proliferation effects of miR-21, while PI3K inhibitor, LY294002, totally attenuated the pro-survival effect of miR-21. These results indicate that miR-21 is efficient in promoting proliferation in c-kit^+^ CSCs, which is contributed by the PTEN/PI3K/Akt pathway. miR-21 holds the potential to facilitate CSC therapy in ischemic myocardium.

## Introduction

Ischemic cardiomyopathy is still the leading cause of deaths worldwide. Despite advances in interventional procedures, such as the catheter-based therapies, the five year mortality rate for myocardial infarction (MI) remain as high as 50% (Mozaffarian et al., 2016). Alternative strategies, such as stem cell-based therapies, are urgently needed (Fisher et al., 2015).

Stem cell-based therapies are efficient in repairing cardiac damage resulted from ischemiareperfusion (I/R) injury (Hong & Bolli, 2014; Sanganalmath & Roberto, 2013). Among the many types of cardiac-derived stem cells being investigated, c-kit^+^ cardiac stem cells (CSCs) appeared to be particularly promising because of their ability of differentiating into three cell types in the myocardium, the cardiomyocytes, smooth muscle cells and endothelial cells (Beltrami et al., 2003). In the past decade, studies have demonstrated that human and rodent c-kit^+^ CSCs promote cardiac regeneration and attenuate heart dysfunction and remodeling after MI in various animal models (Angert et al., 2011; Bearzi et al., 2007; Bolli et al., 2013; Fischer et al., 2009; Linke et al., 2005; Taghavi et al., 2015; Tang et al., 2016; Tang et al., 2010) A recent study showed that the benefits of c-kit^+^ CSCs on ventricular remodeling and dysfunction lasted for more than one year in rats (Tang et al., 2016).

Two Phase I trials, the CADUCEUS and SCIPIO (Malliaras et al., 2014; Bolli et al., 2011) demonstrated the safety and feasibility of cardiac derived stem cells in MI treatment. Despite the minimal cardiomyogenic potential of CSCs (Tang et al., 2016; Van Berlo et al., 2014), reports have demonstrated their potential to promote angiogenesis and decrease cellular apoptosis and necrosis *in vivo*, either by differentiation towards vascular lineages (Tallini et al., 2009) or via secretion of growth factors (Huang et al., 2011) and/or extracellular microRNAs (miRNAs) (Gray et al., 2015).

However, poor engraftment and low viability of CSCs minimizes the percentage survived CSCs and hampers functional improvements and cardiac outcomes (Hu et al., 2011). The very poor survival of donor cells is one of the challenges needed to be overcome before CSC-based therapies become a clinical reality. In mice with MI, it has been shown that >90% of transplanted CSCs die within a week and >95% within five weeks (Hong et al., 2014; Hong et al., 2013). It is apparent that this massive loss of cells limits their effectiveness as a therapy. Strategies to enhance CSC survival after adoptive transfer would have significant therapeutic implications for patients with ischemic heart disease and post-MI heart failure. Strategies to increase cell survival including preconditioning the cells with a variety of techniques, including heat shock of the cells prior to transplantation, forced expression of survival factors in the donor cells, and exposure of cells to pro-survival factors (Haider & Ashraf, 2010; Laflamme et al., 2007; Mohsin et al., 2012). Hu et al. (2011) improved the engraftment of transplanted CSCs and therapeutic efficacy for treatment of ischemic heart disease using a miRNA prosurvival cocktail, which contained miR-21, miR-24 and miR-221.

MicroRNAs are small non-coding RNAs, which inhibit translation or promote mRNA degradation of their target genes (Bartel, 2004; Small, Frost & Olson, 2010). Accumulating evidence indicates that miR-21 plays important roles in tumor growth (Lv, Hao & Tu, 2016), lung tumor cell lines (Xu et al., 2014), skin fibroblasts (Liu et al., 2014) and hepatocyte (Yan-nan et al., 2014) proliferation and cardiac cell growth (Cheng & Zhang, 2010). miRNAs also play critical roles in cardiogenesis and cardiac regeneration (Anton et al., 2011; Fuller & Qian, 2014; Hosoda, 2013; Thomas et al., 2010). Gain-of function studies indicated miR-21 reduces cardiomyocyte apoptosis under oxidative stress (Lv et al., 2016; Wei et al., 2014). Importantly, miRNA expression is capable of controlling CSCs fate and holds the potential of enhancing clinical efficacy of cellular therapy (Hosoda, 2013; Hu et al., 2011). It is reported that miRNAs contribute to CSC differentiation (Hosoda et al., 2011; Van Rooij et al., 2007; Zhao, Samal & Srivastava, 2005). For example, miR-21 not only modulates immunoregulatory function of bone marrow mesenchymal stem cells (BMSCs) through the PTEN/Akt/TGF-*β*1 pathway (Wu et al., 2015), but also enhances human multipotent cardiovascular progenitors therapeutic effects via PTEN/HIF-1*α*/VEGF-A signaling (Richart et al., 2014).

The phosphatase and tensin homolog deleted on chromosome ten (PTEN), which was first found as a tumor suppressor gene, participates in tumor growth, apoptosis, adhesion, invasion and migration (Ciuffreda et al., 2014; Panigrahi et al., 2004). Silencing of PTEN promotes cell proliferation (Gregorian et al., 2009). Pharmacological inhibition of PTEN limits myocardial infarction and improves left ventricular function after MI (Keyes et al., 2010). PTEN works partially through the prosurvival signaling by inhibiting phosphorylation of Akt (p-Akt), which is the active form of Akt (Panigrahi et al., 2004). The up-regulation of PTEN increases apoptosis in cardiomyocytes, whereas its inactivation activates the Akt signaling, reduces apoptosis, and increases survival (Mocanu & Yellon, 2007; Schmid et al., 2004; Schwartzbauer & Robbins, 2001; Wu et al., 2006). It is well documented that PTEN is one of miR-21^′^s target genes (Li et al., 1997; Qi et al., 2015; Stambolic et al., 1998; Wu et al., 2015). Accumulating evidence suggests that miR-21 promotes cell proliferation via PTEN-dependent PI3K/Akt signaling activation in cancer cells (Bai et al., 2011; Di Cristofano & Pandolfi, 2000; Meng et al., 2006; Ou, Li & Kang, 2014; Yan-nan et al., 2014). Gain-of-function of miR-21 can efficiently reduce I/R injury via down-regulation of PTEN (Sayed et al., 2010; Tu et al., 2013). Recently, we found that miR-21 can reduces hydrogen peroxide-induced apoptosis and promotes cell survival in c-kit+ cardiac stem cells *in vitro* through PTEN/PI3K/Akt signaling (Deng et al., 2016).

In this study, by using the gain-of-function experiments of miR-21 in c-kit^+^ CSCs *in vitro*, we provide evidence that miR-21 may accelerate c-kit^+^ CSCs proliferation through the PI3K/PTEN/Akt signaling. This suggests that miR-21 possess the pro-survival ability in c-kit^+^ CSCs *in vivo*. miR-21 could be a potential molecule to facilitate stem cell treatment of ischemic myocardium.

## Materials and Methods

### Animals

Male Sprague-Dawley rats (3-week old, 45–60 g) were purchased from the Third Military Medical University (Chongqing, China), and maintained in Zunyi Medical College. Twelve hours light/dark (8:00 am–8:00 pm light on) cycles were given and they can freely access to rat chow and water. All experimental procedures were performed according to the “Guide for the Care and Use of Laboratory Animals” in China and approved by the Experimental Animal Care and Use Committee of Zunyi Medical College (approval No. 2013032).

### Materials

PE conjugated anti-CD34 and anti-CD45 primary antibodies were from BioLegend (USA). The collagenase type II was from Sigma (St. Louis, MO, USA). Ham’s/F-12 medium and fetal bovine serum (FBS) were purchased from HyClone (Logan, UT, USA). Trypsin was purchased from Gibco (Billings, MT, USA). Penicillin and streptomycin were from Sorlabio (Beijing, China). Fibroblast growth factor was from Peprotech (Rocky Hill, NJ, USA). Leukocyte inhibitory factor was product of Gibco. Rabbit anti-rat c-kit^+^ primary antibody was supplied by Biorbyt (Cambridge, UK). M-280 beads conjugated with sheep anti-rabbit secondary antibody were from Dynal Biotech (Hovik, Norway). miR-21 mimics and the negative control scramble were synthesized by RIBOBIO (Guangzhou, China). Lipofectamine 2,000 was from Invitrogen (Carlsbad, CA, USA). Primers, miRNA reverse transcript kit and qRT-PCR kit were from Sangon Biotech (Shanghai, China). Anti-*β*-Actin, anti-PTEN, anti-BrdU, anti-P-Akt, anti-Akt primary antibody, and other secondary antibodies were obtained from Boster bio (Wuhan, China). EdU (5-ethynyl-29-deoxyuridine) cell proliferation detecting kit was from RIBOBIO (China). Cell cycle detecting kit was from KeyGEN (Nanjing, China). LY294002 (PI3K inhibitor) was from Beyotime (Jiangsu, China). Phen (PTEN inhibitor) was product of Merck (Darmstadt, Germany). The unlisted reagents were of analytical grade.

### c-kit^+^ cells isolation, purification and identification

CSCs were isolated (Choi et al., 2013) and purified (Kazakov et al., 2015) using previously published methods, with some modifications. Breifly, rats were deeply anesthetized with sevoflurane, then the atrial appendage was sliced and digested with 0.1% collagenase type II (Sigma). After about 40 min digestion at 37 °C, cells were collected by sedimentation at 1,200 rpm for 5 min (min). Then cells from atrial appendage were incubated in a humidity chamber in Ham’s F12 medium containing 10% FBS, 1% penicillin and streptomycin, 1% L-glutamine, 20 ng/mL human recombinant fibroblast growth factor, 20 ng/mL leukocyte inhibitory factor, 10 ng/mL epidermal growth factor (EGF). When cells confluence reached >90%, they were suspended by trypsinization. Then CSCs were incubated with rabbit anti-c-kit antibody (1:250 in F12 medium) for 1 h (h), and sorted with anti-rabbit secondary antibody conjugated 2.8 µm magnetic beads (Dynal Biotech, Hovik, Norway) in 30 min as instructed by the manufacturer’s protocols. The purified c-kit^+^ CSCs were cultured in aforementioned F12 medium. Flow cytometry was used to confirm the expression patterns of CSCs markers. Cells were incubated with fluorochrome-conjugated primary antibodies: anti-CD34-PE, anti-CD45-PE, and anti-c-kit primary antibody and anti-c-kit IgG- allophycocyanin (APC) secondary antibody (all from BioLegend, San Diego, CA, USA).

### miR-21 mimics transfection and the detection of miR-21 level in CSCs

For the miR-21 gain-of-function experiments, 2 µg miR-21 mimic or its control scramble were added in 1.5 mL F12 medium in 6-well plates with 5 µL transfection reagent Lipofectamine 2,000 (Invitrogen), and incubated with c-kit^+^ CSCs for 48 h according to the manufacturer’s instructions. Real-time PCR was used to detect miR-21 expression change.

### Cell proliferation and viability detection with CCK-8 kit

Adult cardiomyocytes’ viability was detected with Cell Counting Kit-8 (CCK-8, Beyotime, China) as previously reported (Cao et al., 2015). The same amount of CSCs were seeded into 24-well plates and incubated with miR-21 mimics or its scramble for 24, 48 or 72 h, then 30 µL WST-8 solution was added into F-12 medium to form a 3% WST-8 solution (final concentration). After 1 h incubation, the mixture’s optical density (OD) values were detected at 450 nm wavelength.

### Immunofluorescence of c-kit

To characterize the purity of isolated CSCs, immunocytochemistry was used to verify c-kit expression on purified cells as reported elsewhere (Elisabetta et al., 2013). Cells were fixed with 4% paraformaldehyde, then blocked with 10% goat serum before incubated with anti-c-kit antibody. c-kit^+^ CSCs were subsequently incubated with FITC-conjugated secondary antibody. After washing, the nuclei were counterstained with DAPI. The immunofluorescence photos were taken with a fluorescence microscope (Olympus, Japan).

### Proliferation detection with EdU assay

To detect proliferation of c-kit^+^ CSCs, the EdU assay kit was employed according to the manufacturer’s instructions. Briefly, c-kit^+^ CSCs were cultured in triplicate in 96-well plates and were transfected with 50 nM of miR-21 mimics or its control RNA for 48 h. The cells were then exposed to 50 mM EdU for additional 4 h at 37 °C. Then, CSCs were fixed with 4% formaldehyde for 15 min and treated with 0.5% Triton X-100 for 20 min at room temperature. Then, cells were incubated with Apollo cocktail then the DNA contents of CSCs were stained with Hoechst for 30 min and visualized under a fluorescent microscope (Olympus, Tokyo, Japan).

### Cell cycle assay

Cell cycle was determined by flow cytometry. Briefly, CSCs were cultured in 6-well plates and transfected with 50 nM of miR-21 mimics or its scramble for 48 h. The c-kit^+^ CSCs were then fixed in 70% ethanol for 24 h, followed by propidium iodide (PI) staining. The cell cycle phases were analyzed using a flow cytometry instrument (BD, FACS Calibur; San Jose, CA, USA).

### Reverse transcription and Real-Time PCR of miR-21 and PTEN

mRNA and miRNA levels were determined by using quantitative RT-PCR as previously reported (Cao et al., 2015; Cheng et al., 2009). Briefly, RNAs from CSCs were isolated with the TRIzol (Invitrogen) method. RT-PCR was performed on cDNA generated from 3 µg of total RNA with a cDNA synthesis kit (TaKaRa, Tokyo, Japan) according to the manufacturer’s protocol. RT-qPCR was performed with the CFX Connect Real-Time system (Bio-Rad, USA) using a SYBR green PrimScript RT kit (TaKaRa) based on the manufacturer’s instructions. The PCR conditions included pre-denaturing at 95 °C for 30 s followed by 40 cycles of denaturation at 95 °C for 10 s and combined annealing/extension at 58 °C for 30 s. All the mRNA expression levels were calculated based on the comparative quantification method (2^−ΔΔCT^). The U6 and *β*-actin were used as internal controls for miR-21 and PTEN mRNA quantitation respectively.

### Western blot

Western blot analysis of total protein from c-kit^+^ cell lysis was performed as described previously (Cao et al., 2016). The protein extracts were separated by SDS-polyacrylamide gels electrophoresis (SDS-PAGE) and transferred to PVDF membranes. After overnight blocking in nonfat milk solution, membranes were probed with anti -PTEN, -phospho-Akt, -Akt, -BrdU, -*β*-actin or -GAPDH primary antibodies. PVDF membranes were incubated with horseradish peroxidase-conjugated secondary antibodies for 1 h and then enhanced chemiluminescence (Amersham Biosciences, Sunnyvale, CA, USA). Immunoreactivity was visualized by a ChemiDoc MP system (Bio-Rad). Protein levels were normalized to *β*-actin or GAPDH.

### Statistical analysis

Data are presented as mean ± SD. All data were analyzed by the Student’s *t*-test or by one-way ANOVA followed by LSD or Dunnett’s T3 post-hoc test for multiple comparisons. A P value of less than 0.05 was considered to be statistically significant. Data analyses were carried out using SPSS (v.19.0, IBM, USA).

## Results

### Isolated c-kit^+^ CSCs

**Figure 1:**
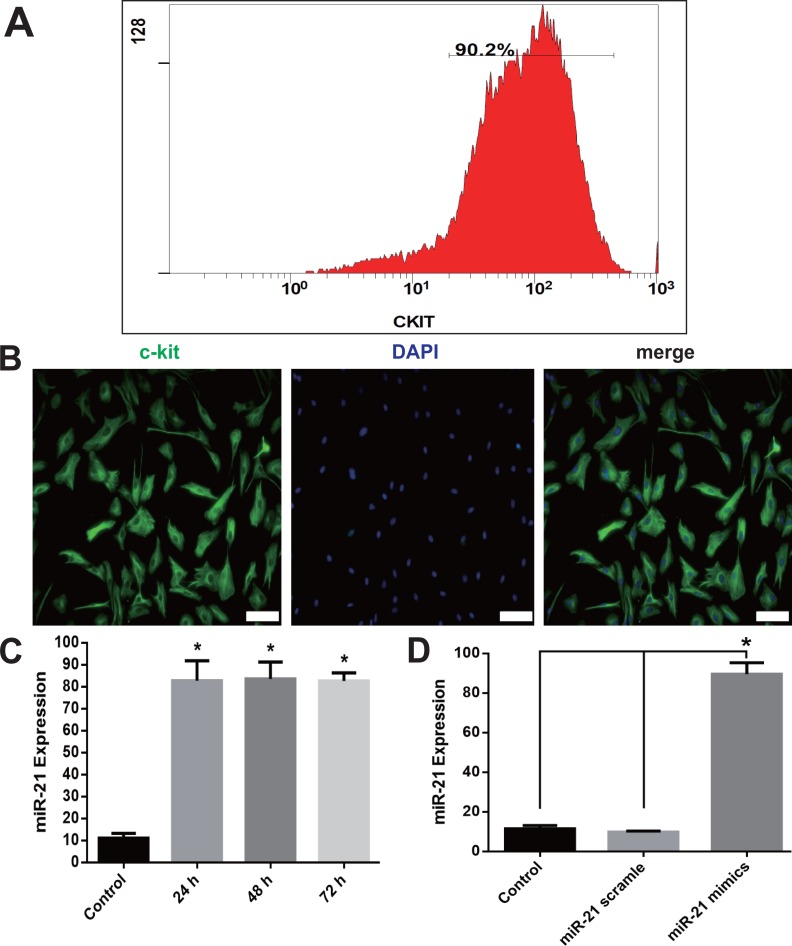
c-kit^+^ CSCs isolation and overexpression of miR-21in CSCs. After isolation from rat atrial appendage, cells were purified by a combined use of c-kit antibody and magnetic beads conjugated with secondary antibody. Flow cytometry showed c-kit^+^ cells were more than 90% (A). (B) Purified cells were double stained by c-kit (green) and DAPI (blue), and observed under a fluorescence microscope (Olympus). Bar = 50 µm. (C) Cultured CSCs were treated with miR-21 mimics for 24, 48 or 72 h before miR-21 RT-PCR detection. miR-21 mimics significantly increased miR-21 but no difference was detected among the three time points. *, *P* < 0.05 compared with Control. (D) CSCs were incubated with miR-21 mimics or its negative control scramble for 48 h. miR-21 mimics significantly increased miR-21 level in c-kit^+^ CSCs. *, *P* < 0.05. *n* = 3 in each group.

c-kit^+^ CSCs were isolated from rat atrial appendage and purified using anti-rabbit secondary antibody conjugated magnetic beads. Flow cytometry showed that 90.2% of cells were c-kit positive after the purification (Fig. 1A). Purified cells were stained with anti-c-kit antibody, and counterstained with DAPI to visualize the nuclei. The immunofluorescence staining also showed a high percentage of double-staining of c-kit^+^ and DAPI (Fig. 1B).

### Transfection of CSCs with miR-21 mimics increased miR-21 expression

RT-PCR of miR-21 showed a significant increase of miR-21 when cells were transfected with miR-21 mimics 48 h later (*P* < 0.05 compared with Control or Scramble group, Figs. 1C–1D). The up-regulation of miR-21 was stable at 72 h, and no difference was detected among 24, 48 and 72 h group (Fig. 1C). We choose 48 h as the incubation time in the subsequent experiments.

### miR-21 increased proliferation in CSCs

**Figure 2:**
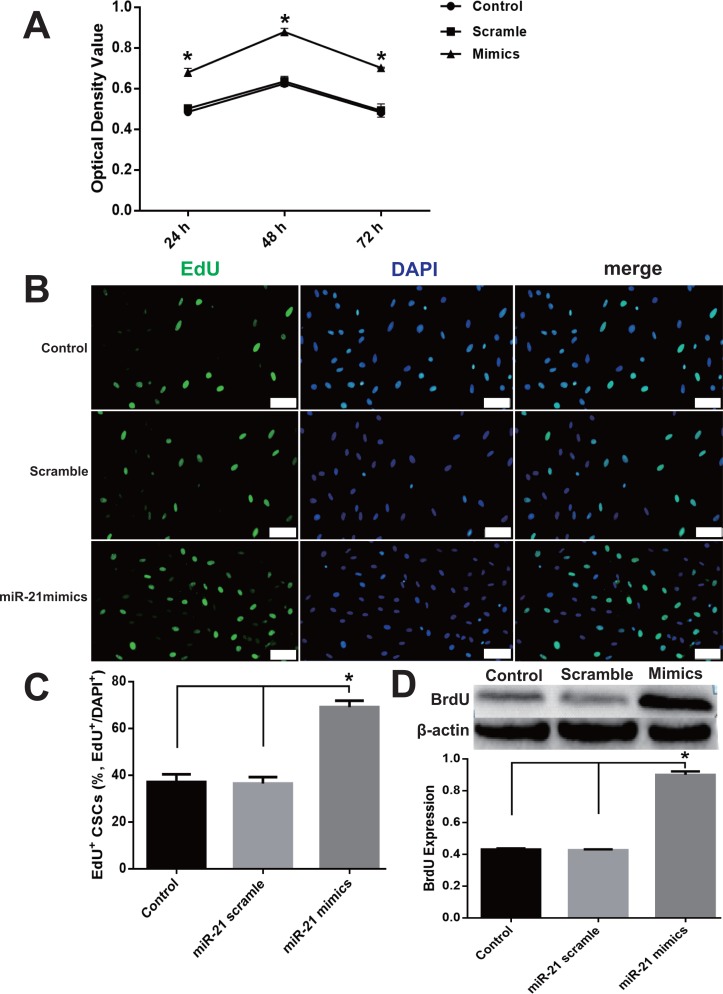
miR-21 effect of on CSC proliferation. (A) Cultured CSCs were treated with miR-21 mimics or its negative control scramble for 24 h, 48 h or 72 h respectively. Cell proliferation and viability were detected with CCK-8 assay. miR-21 mimics remarkably increased proliferation of c-kit^+^ CSC at the three time points (with *P* values < 0.05) and 48 h was the peak point of cell viability. *n* = 3. (B) c-kit^+^ CSCs were double stained by c-kit (green) and DAPI (blue), and observed under a fluorescence microscope (Olympus). Bar = 50 µm. DAPI = propidium iodide. (C) The statistics of EdU positive CSCs from immunofluorescence in (B). *n* = 6 in each group. (D) miR-21 mimics’s in-fluences on BrdU expression, which was detected with immune blotting. miR-21 mimics dramatically increased the expression of BrdU compared with Control or Scramble group. *n* = 3. *, *P* < 0.05.

The pro-proliferation effect of miR-21 was detected with CCK-8 and EdU assays and immunoblotting of BrdU. miR-21 significantly increased cell proliferation compared with the negative control scramble group (Fig. 2), which is evidenced by the increased OD value in CCK-8 experiments (Fig. 2A) and a larger proportion of EdU positive CSCs in EdU assay analysis (Figs. 2B–2C). In addition, the BrdU expression was markedly increased, in the miR-21 mimics group compared with Control or Scramble group (Fig. 2D).

### miR-21 decreased PTEN protein expression

**Figure 3:**
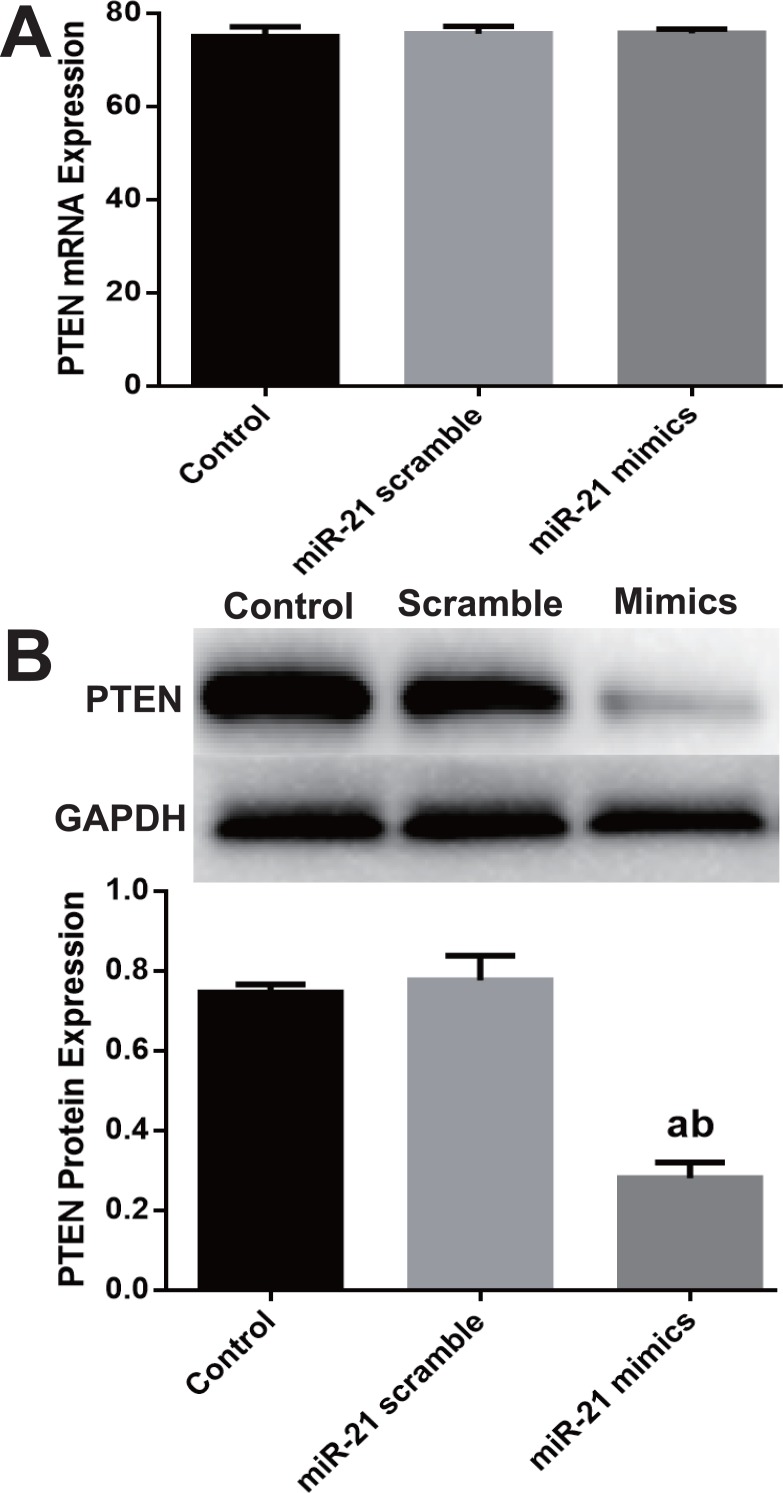
miR-21 effect of on PTEN expression in CSCs. Cultured CSCs were treated with miR-21 mimics or its negative control scramble for 48 h, then cells were harvested and subjected to RT-PCR or Western blot. PTEN mRNA of Control, scramble treated or miR-21 mimics treated CSCs showed no significant difference (A), but PTEN protein dramatically decreased after miR-21 mimics treatment (B). a, *P* < 0.05 compared with Control; b, *P* < 0.05 compared with Scramble. *n* = 3 in each group.

Although PTEN was extensively reported as one of miR-21^′^s target genes in many cell types, Western blot was employed to verify miR-mimic’s effect on PTEN expression in c-kit^+^ CSCs. mRNA level of PTEN didn’t change (Fig. 3A), while PTEN protein significantly down-regulated in mimics group as compared with Control or Scramble group (*P* < 0.05, Fig. 3B).

### miR-21 increase proliferation of c-kit^+^ CSCs via the PTEN/PI3K/Akt pathway

**Figure 4:**
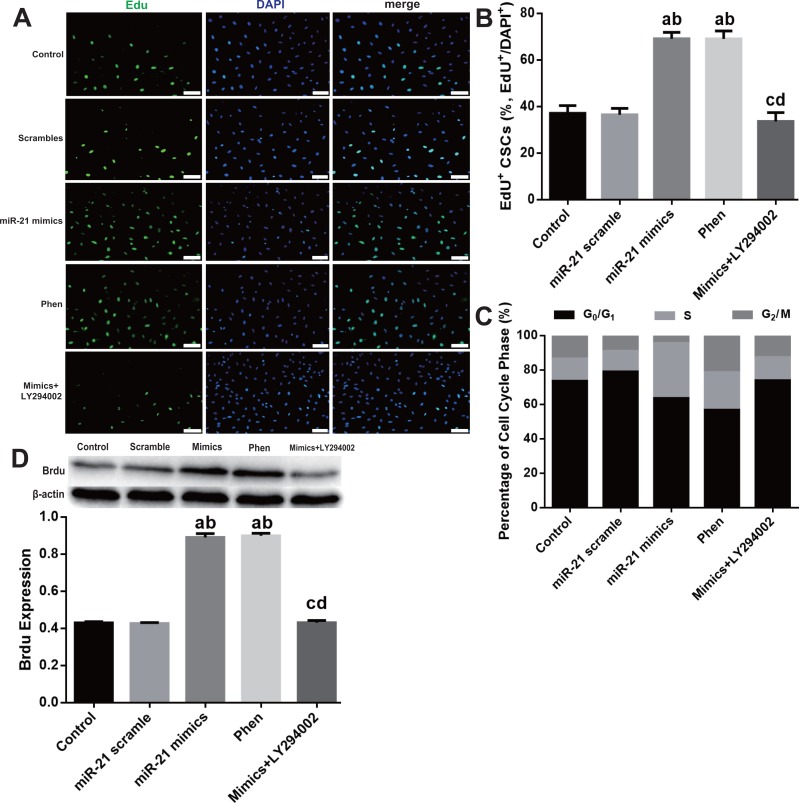
PTEN/PI3K/Akt pathway’s contribution in miR-21 induced proliferation in c-kit^+^ CSCs. Cultured c-kit^+^ CSCs were treated with miR-21 mimics for 48 h before subjected to EdU immunofluorescence (A–B), flow cytometry (C) or Western blot (D). To test the contribution of PTEN/PI3K/Akt signaling, PTEN and PI3K were inhibited with Phen or LY294002 respectively. (A) c-kit^+^ CSCs were double stained by EdU (green) and DAPI (blue), and observed under a fluorescence microscope (Olympus). Bar = 50 µm. DAPI = propidium iodide. (B) The statistics of EdU positive CSCs from immunofluorescence in (A). *n* = 6 in each group. (C) Flow cytometry was employed to detect cell cycle profiles in CSCs underwent different treatments miR-21 mimics or Phen increased the proportion of S phase CSCs compared with Control or scramble treated groups. *n* = 3. (D) PTEN/PI3K/Akt pathway’s influences on BrdU expression, which was detected with immune blotting. Just like miR-21 mimics’ effect on BrdU, when PTEN was inhibited by Phen, there was notably increase of BrdU compared with Normal or Scramble group. When PI3K was inhibited by LY294002, there was notably decrease of BrdU in mimics+LY294002 group compared with mimics group in CSCs. *n* = 3 in each group. a, *P* < 0.05 compared with Control; b, *P* < 0.05 compared with Scramble; c, *P* < 0.05 compared with miR-21 mimics group; d, *P* < 0.05 compared with Phen group.

To study the mechanisms responsible for miR-21 mediated pro-proliferation effects in c-kit^+^ CSCs, we blocked PTEN and PI3K with their specific inhibitors Phen or LY294002 respectively. Phen administration increased proliferation of CSCs just like the effect of miR-21 mimics, while LY294002 partially reversed the pro-proliferation effect of miR-21 mimics (all *P* < 0.05 Figs. 4A–4B). Flow cytometry was employed to detect cell cycle profiles in CSCs underwent different treatments miR-21 mimics or Phen increased the proportion of S phase CSCs compared with Control or scramble treated groups (Fig. 4C). Just like miR-21 mimics’ effect on BrdU, when PTEN was inhibited by Phen, there was notably increase of BrdU compared with Control or Scramble group. When PI3K was inhibited by LY294002, there was notably decrease of BrdU in mimics+LY294002 group compared with mimics group in CSCs (Fig. 4D).

**Figure 5:**
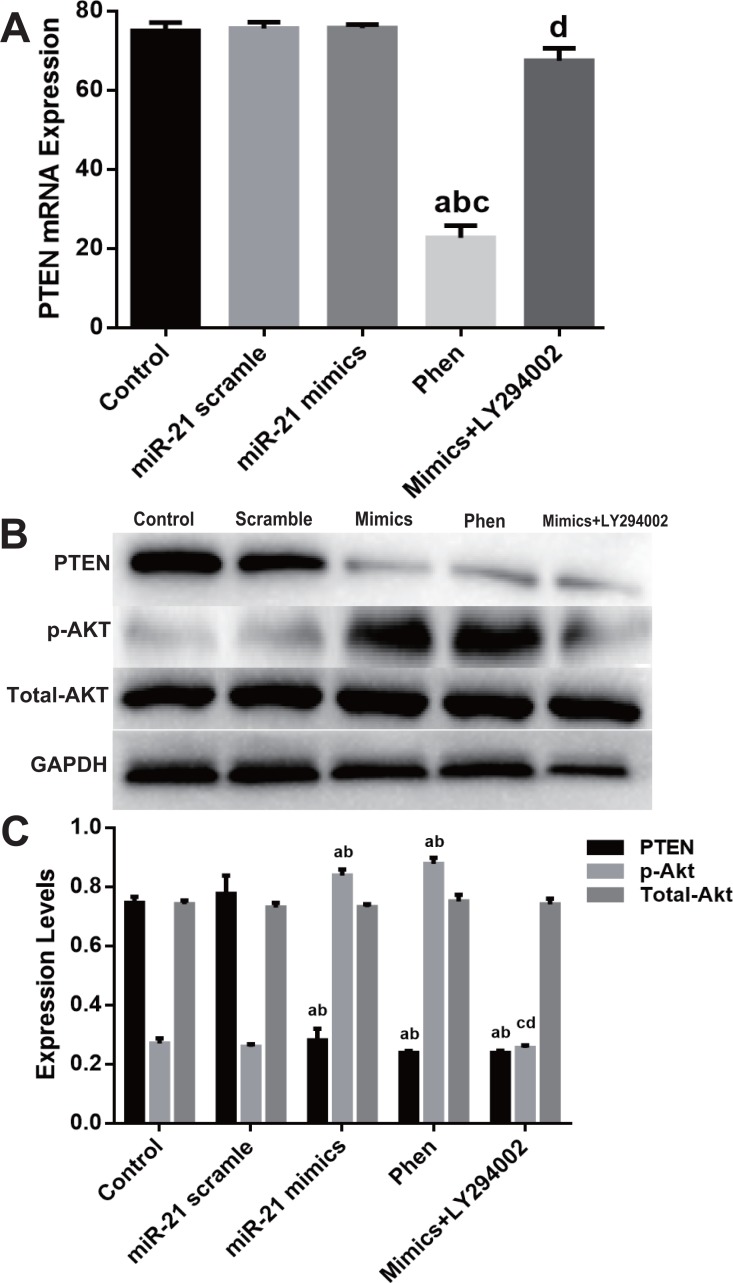
Expression change of PTEN/PI3K/Akt pathway in the process of miR-21 mimics induced proliferation in c-kit^+^ CSCs. Cultured CSCs were treated with miR-21 mimics for 48 h before the subsequent procedures. To test the contribution of PTEN/PI3K/Akt signaling to miR-21 mimics’s pro-proliferation effects in c-kit^+^ CSCs, PTEN and PI3K were inhibited with Phen or LY294002 respectively. (A) RT-PCR was carried out to detect miR-21 mimics’s effects on PTEN expression at the mRNA level, which showed no change between Control, miR-21 scramble, miR-21 mimics and miR-21 mimics+ LY294002 group, while Phen resulted in a significant down-regulation of PTEN compared with the other groups. (B–C) Western blot was carried out to detect miR-21 mimics’s effects on PTEN protein expression, which showed that miR-21 mimics significantly down-regulated PTEN protein in miR-21 mimics group compared with the scramble group. In addition, both Phen treatment and miR-21 mimics incubation increased p-Akt level, while PI3K inhibitor LY294002 decreased p-Akt level dramatically (*P* < 0.05). a, *P* < 0.05 compared with Control; b, *P* < 0.05 compared with miR-21 scramble group; c, *P* < 0.05 compared with miR-21 mimics group; d, *P* < 0.05 compared with Phen group. *n* = 3 in each group. p-Akt = phosphor-Akt.

Molecular detection of PTEN/PI3K/Akt pathway displayed that Phen efficiently decreased mRNA level of PTEN while LY294002 showed little effect (Fig. 5A). However, Phen significantly increased p-Akt and LY294002 reversed miR-21 mimics’ effect on p-Akt level (Figs. 5B–5C).

## Discussion

Stem cell based therapy is promising for the prevention or treatment of ischemic cardiomyopathy (Mozaffarian et al., 2016). c-kit^+^ CSCs is one of the most promising stem cell types (Nigro et al., 2015). Nevertheless, poor engraftment minimizes the survival rate of injected stem cells that contribute to heart functional improvement (Hu et al., 2011). miRNAs hold the potential to improve engraftment and functional outcomes of CSC transplantation (Hosoda, 2013; Hu et al., 2011). Studies have shown that miR-21 protects myocardium from ischemic injury (Cheng et al., 2009). miR-21 also protects BMSCs (Lv et al., 2016) and cardiomyocytes (Cheng et al., 2009; Wei et al., 2014) from H_2_O_2_ induced cell damage, such as the apoptosis and necrosis. We also found that miR-21 reduces hydrogen peroxide-induced apoptosis and promotes cell survival in c-kit+ cardiac stem cells *in vitro* through PTEN/PI3K/Akt signaling (Deng et al., 2016). However, it is not known whether miR-21 can promote proliferation in c-kit^+^ CSCs. Additionally, the underlying molecular mechanisms between miR-21 and CSC proliferation need to be elucidated.

In this study, we established an *in vitro* miR-21 gain-of-function model to test miR-21’s effect on proliferation and the involvement of PI3K/Akt signaling pathway. The pro-proliferation effect of miR-21 was detected with CCK-8 and EdU assays and immunoblotting of BrdU. miR-21 significantly improved cell proliferation parameters in the three kind of experiments. Flow cytometry was employed to detect cell cycle profiles in CSCs after miR-21 mimics transfection. miR-21 mimics increased the proportion of S phase CSCs. These results indicate that miR-21 may be a pro-survival factor in c-kit^+^ CSCs *in vitro*.

PTEN has been extensively reported as one of target genes of miR-21, but it is not confirmed in c-kit^+^ CSCs to our knowledge. In many cell types, e.g., hepatocytes, cardiomyocytes and cancer cells, miR-21 mediates the expression of PTEN (Lv, Hao & Tu, 2016; Qi et al., 2015; Tu et al., 2013). We hypothesized that PTEN is the target gene of miR-21 in c-kit^+^ CSCs too. PTEN expression was directly examined after up-regulation of miR-21 and the Western blot results confirmed our assumption. miR-21 over-expression caused significant down-regulation of PTEN protein although the mRNA did not change much

The PI3K/Akt pathway participates in inhibiting apoptosis and promoting cell proliferation (Mark, 2007). The activation of Akt significantly protects cells from oxidation induced cell apoptosis (Suk Ho et al., 2010; Yang et al., 2006). It was reported that miR-21 acts via the PI3K/Akt pathway by the down-regulation of PTEN (Qi et al., 2015), which is the upstream of PI3K/Akt pathway, but this effect has not been investigated in c-kit^+^ CSCs. To study whether the PTEN/PI3K/Akt signaling is responsible for miR-21 mediated pro-proliferation effect, we blocked PTEN and PI3K with their specific inhibitor Phen or LY294002 respectively, and examined the phosphorylation of Akt. Just like the pro-proliferation effects of miR-21, Phen administration increased proliferation in c-kit^+^ CSCs. PI3K blocker LY294002 partially reversed pro-survival effects of miR-21 mimics. Furthermore, both Phen and miR-21 mimics increased p-Akt level, while PI3K inhibitor LY294002 decreased p-Akt level dramatically, which suggests that Akt is the downstream of PI3K and Phen.

In the present study, PI3K inhibitor nearly completely offset the pro-proliferation effects of miR-21. At the same time, PTEN inhibitor Phen increased proliferation of c-kit^+^ CSCs to a great extent equal to miR-21. This indicated miR-21’s pro-proliferation effect was mostly achieved via PTEN-PI3K signaling, and just PTEN inhibition with Phen can reproduce pro-survival effect of miR-21. However, what should be realized is that miR-21 targets more than one genes and PI3K/Akt is not the only downstream pathway of PTEN. For example, miR-21 protects cardiac myocytes from the H_2_O_2_-induced injury by targeting PDCD4 gene (Cheng et al., 2009). In addition, miR-21 targets the tissue inhibitor of metalloproteinase-3 (TIMP-3) gene to influence glioma migration and invasion (Galina et al., 2008). PTEN also enhances human multipotent cardiovascular progenitors therapeutic effects via miR-21 initiated PTEN/HIF-1*α*/VEGF-A signaling (Richart et al., 2014).

In conclusion, our data reveal that miR-21 promotes proliferation in c-kit^+^ CSCs partially through the PTEN/PI3K/Akt pathway. The present study demonstrates that miR-21 is a pro-survival molecule for c-kit^+^ CSCs. It also indicates that modification on miRNA expression may be able to enhance the clinical efficacy of cellular therapy.

We must confess some shortcomings of this study. The direct link to any favorable effect of miR-21 on CSC proliferation in clinical trials is limited. *In vivo* studies are warranted to further confirm miR-21 and the PTEN/PI3K/Akt pathway’s effects on survival of c-kit^+^ CSCs. Besides, the luciferase assay could be a more direct and relevant way to confirm that PTEN is the target gene of miR-21.

##  Supplemental Information

10.7717/peerj.2859/supp-1 Data S1 Raw data

## Additional Information and Declarations

### Data Availability

The following information was supplied regarding data availability: The raw data has been supplied as a Supplemental Dataset.
